# Food order affects blood glucose and insulin levels in women with gestational diabetes

**DOI:** 10.3389/fnut.2024.1512231

**Published:** 2024-12-24

**Authors:** Ria Murugesan, Janardanan Kumar, Shubhashree Thiruselvam, Kakithakara Vajravelu Leela, K. Geetha, Abhishek Satheesan, Venkata Chaithanya, Matcha Angelin

**Affiliations:** ^1^Department of Microbiology, SRM Medical College Hospital and Research Centre, SRM IST, Kattankulathur, Tamil Nadu, India; ^2^Department of General Medicine, SRM Medical College Hospital and Research Centre, SRM IST, Kattankulathur, Tamil Nadu, India; ^3^Department of Obstetrics and Gynaecology, SRM Medical College Hospital and Research Centre, SRM IST, Kattankulathur, Tamil Nadu, India; ^4^Department of Clinical Nutrition and Dietetics, SRM Medical College Hospital and Research Centre, SRM IST, Kattankulathur, Tamil Nadu, India

**Keywords:** gestational diabetes mellitus, food order, regular diet, glycemic control, type 2 diabetes

## Abstract

**Background:**

Gestational diabetes mellitus (GDM) poses significant risks to both maternal and fetal health, including a heightened risk of developing type 2 diabetes (T2DM) in the future. Effective management often involves dietary changes, such as food-order, where vegetables are consumed first, followed by proteins, and then carbohydrates last. This study investigates whether food sequence improves glycemic control in women with GDM by slowing carbohydrate absorption, reducing glucose spikes, and enhancing insulin sensitivity.

**Methods:**

Twenty-five women with GDM participated in a four-week trial with three phases: baseline measurement (week 0), phase 1 (regular diet), and phase 2 (food-order) intervention. Primary outcomes were blood glucose and serum insulin levels, measured at fasting, 1-h, and 2-h postprandial intervals during each phase. In phase 1 (weeks 0–2), participants followed their usual diet. In phase 2 (weeks 2–4), the same participants followed a food-order regimen: vegetables first, then proteins, and carbohydrates last. Customized meal plans for vegetarians and non-vegetarians were provided. Participants were monitored via a mobile application (Jotform) for adherence. Follow-up blood glucose and insulin were measured before, and 60 and 120 min after, consuming a standardized meal (339 kcal, 16.4 g protein, 56.1 g carbohydrates, 3.4 g fat) in the clinic.

**Results:**

The food-order intervention resulted in a significant reduction in postprandial blood glucose by 5.87% (*p* = 0.001) at 60 min and 6.06% (*p* = 0.001) at 120 min. Also, Serum insulin levels decreased by 8.13% (*p* = 0.001) at 60 min and 11.10% (*p* = 0.001) at 120 min, compared to the regular diet. These results suggest improved metabolic control and insulin sensitivity.

**Conclusion:**

Prioritizing vegetables before protein and carbohydrates improves glycemic control and insulin sensitivity in women with GDM. This simple strategy helps regulate blood glucose and may reduce the long-term risk of T2DM. It offers a practical approach to managing GDM, but further research with larger cohorts and longer interventions is needed to assess its long-term effects.

**Clinical trial registration:**

https://ctri.nic.in/Clinicaltrials/regtrial.php?modid=1&compid=19&EncHid=81473.12293, identifier CTRI/2024/01/061220.

## 1 Introduction

Gestational diabetes mellitus (GDM) is a condition that develops during pregnancy when the body does not produce sufficient insulin to manage elevated blood sugar levels. It is characterized by glucose intolerance that typically emerges either at the onset or during pregnancy (1). GDM can have lasting impacts, raising the risk of type 2 diabetes mellitus (T2DM) for both the mother and child, also contributing to childhood obesity (2). To manage gestational diabetes effectively, lifestyle modifications are essential (3). Individuals with GDM should focus on creating a balanced diet and adopting long-term healthy eating habits, especially reducing intake of carbohydrates (CHO) which is the primary nutrient, affecting postprandial blood glucose levels. Both the amount and type of CHO consumed play a pivotal role in glucose management (4). The concept of food order refers to the sequence in which different types of foods, such as vegetables, proteins, and carbohydrates, are consumed during a meal. This practice is grounded in the belief that food arrangement may maximize health benefits, enhance digestion, and improve blood glucose and insulin responses. Studies suggested that eating vegetables followed by proteins and finishing off with carbohydrates can lower glycemic and insulin levels (5–7). Foods with a high glycemic index (GI) lead to quick spikes in blood glucose levels, whereas foods with a low GI produce slower, more gradual increases in blood sugar. Vegetables generally have a low GI due to their high fiber content, which slows down the digestion and absorption of CHO (8). This leads to a more steady and gradual increase in blood glucose levels. Moreover, consuming proteins and/or fats before carbohydrates has been shown to stimulate the release of glucagon-like peptide-1 (GLP-1), a hormone crucial for maintaining glucose homeostasis. GLP-1 enhances glucose-induced insulin secretion and suppresses glucagon secretion, playing an important role in regulating blood sugar levels (9). Additionally, GLP-1 also functions as a satiety hormone, contributing to reduced appetite and increased fullness (10). The timing of macronutrient intake specifically the consumption of protein and fat before CHO, enhances GLP-1 secretion, which in turn slows gastric emptying and improves postprandial glucose excursions.

Although food order has been studied in the context of managing blood glucose levels in individuals with T2DM (5, 7, 11), its potential effects on women with GDM have not been adequately explored much. While both T2DM and GDM are related to impaired glucose metabolism, the physiological and hormonal changes during pregnancy make GDM a distinct condition that requires targeted management strategies. Existing research on food order predominantly focuses on its role in T2DM, and there is a notable gap in understanding whether the same principles apply to pregnant women with GDM. This gap is critical because the dietary needs and glucose responses in pregnant women with GDM may differ from those in individuals with established diabetes. Specifically, pregnancy-induced hormonal changes (1), such as increased levels of insulin resistance (2) and altered glucose metabolism (6), may influence how women with GDM respond to food order and dietary modifications. This study seeks to address this significant gap by investigating the effects of a specific food-ordering regimen—consuming vegetables first, followed by proteins, and finishing with carbohydrates on blood glucose and insulin levels in women with GDM. The novelty of this research lies in its exploration of a straightforward dietary intervention that could have profound implications for managing GDM. The potential to improve glycemic control through such simple dietary modifications offers a promising and accessible approach for women with GDM. Furthermore, this study integrates the use of a mobile health application to track dietary adherence and provide feedback, combining a traditional dietary intervention with modern technology to enhance patient compliance. The primary objective of this study is to assess whether modifying the sequence in which foods are consumed can lead to improved glycemic control in women with GDM. If successful, this approach may contribute to better maternal health outcomes during pregnancy, as well as reduce the long-term risk of developing T2DM. By addressing the specific needs of women with GDM, this research aims to inform future dietary guidelines and offer a practical, evidence-based strategy for managing GDM in clinical practice. The findings from this study could offer valuable insights for healthcare providers and dietary counsellors, promoting healthier eating patterns that support both maternal and fetal health during pregnancy.

## 2 Methodology

This study was conducted at SRM Medical College Hospital and Research Centre, SRM IST, Chennai, India.

### 2.1 Ethical considerations

The clinical study registered in CTRI (Clinical Trial Registry India) with CTRI number CTRI/2024/01/061220, also approved by Institutional Ethical Clearance (Number: 8707/IEC/2023).

### 2.2 Study participants

Twenty-five women with gestational diabetes mellitus were recruited from an obstetric clinic for the study, with an average age of 26.18 ± 2.29 years, a BMI of 30.26 ± 3.14 kg/m^2^, and a gestational age of 26.52 ± 0.72 weeks. The mean HbA1c was 6.1 ± 0.42%, while other metabolic and cardiovascular parameters included systolic blood pressure of 114.6 ± 11.02 mmHg, diastolic blood pressure of 74.2 ± 7.31 mmHg, fasting blood glucose (FBG) of 101.88 ± 5.65 mg/dL, 2-h postprandial blood glucose (2 h PPBG) of 130.76 ± 3.44 mg/dL, total cholesterol of 212.64 ± 10.02 mg/dL, triglycerides of 178.64 ± 47.59 mg/dL, LDL-c of 144.08 ± 5.67 mg/dL, and HDL-c of 43.88 ± 5.84 mg/dL. None of the participants had a history of diabetes prior to their diagnosis with GDM. Informed consent was obtained from all participants, and an information sheet outlining the overall intervention was provided and explained to each individual (Table 1).

**Table 1:** Baseline characteristics of study participants.

GDM (25)	Study participants
Age	26.18 ± 2.29
Gestational week	26.52 ± 0.72
Height (cm)	156.54 ± 4.64
Weight (kg)	74.02 ± 9.63
BMI	30.26 ± 3.14
HbA1c	6.1 ± 0.42
Systolic (mmHg)	s114.6 ± 11.02
Diastolic (mmHg)	74.2 ± 7.31
FBG (mg/dL)	100.68 ± 4.6
2 h PPBG (mg/dL)	130.76 ± 3.4
Total cholesterol (mg/dL)	212.64 ± 10.02
Triglycerides (mg/dL)	178.64 ± 47.59
LDL-c (mg/dL)	144.08 ± 5.67
HDL-c (mg/dL)	43.88 ± 5.84

Data are represented as mean ± SD. cm, centimeter; kg, kilogram; mmHg, millimeters of mercury, FBG, fasting blood glucose; PPBG, postprandial blood glucose; HbA1c, glycated hemoglobin; mg/dL, milligrams per deciliter; LDL-c, low-density lipoprotein cholesterol; HDL-c, high-density lipoprotein cholesterol.

### 2.3 Study design

**Figure 1:**
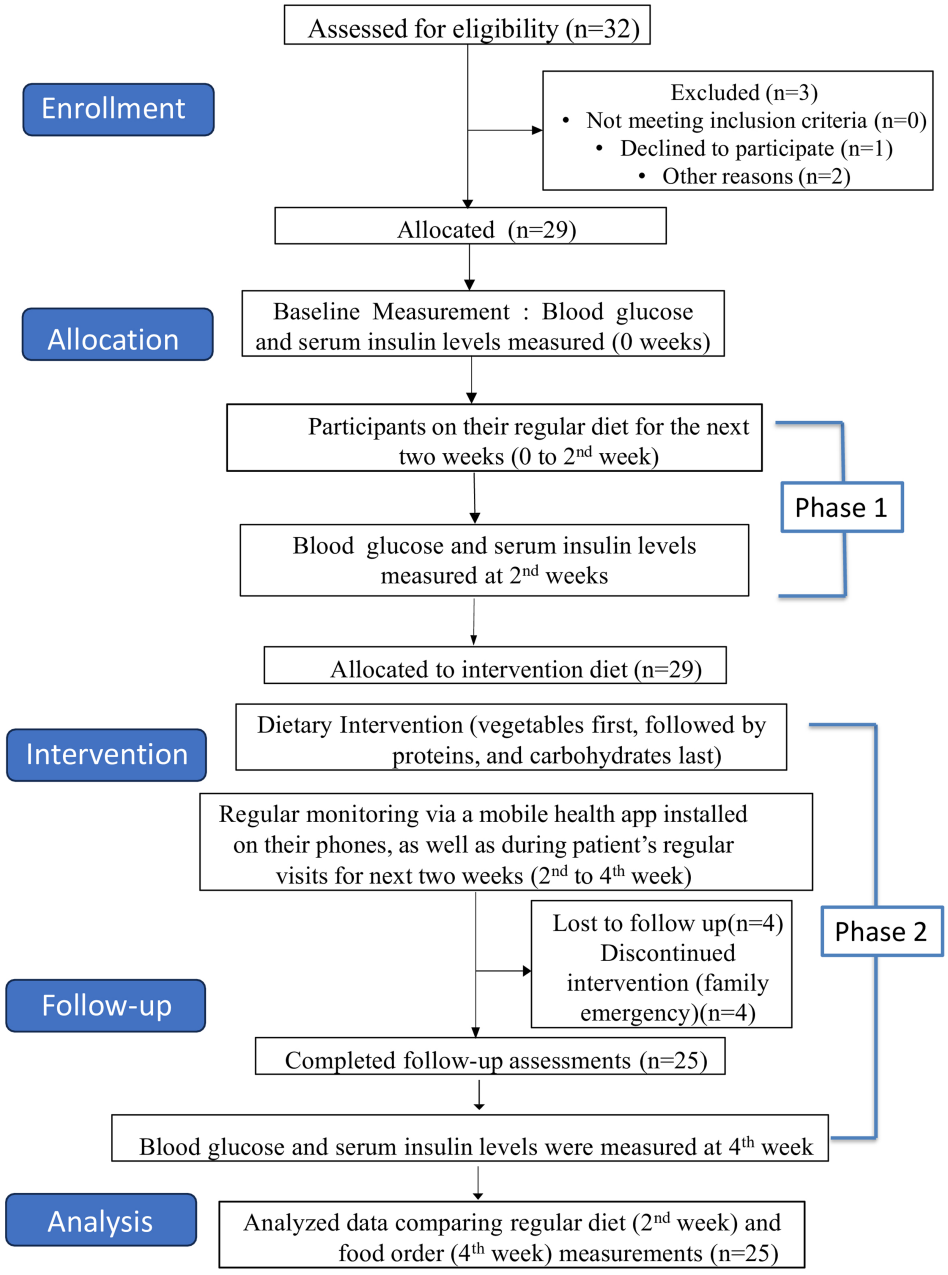
CONSORT flow diagram.

This study employed an open-label, interventional trial design to assess the effects of a dietary intervention (food order) on glycemic control in 25 women diagnosed with GDM. The participants underwent a four-week trial, consisting of baseline measurements and two distinct phases (Figure 1).

#### 2.3.1 Baseline measurement (0 week)

After a 12-h overnight fast, participants consumed their usual regular meal. Blood glucose and serum insulin levels (at fasting, 1 h, 2 h), other relevant clinical parameters, such as BMI, lipid profile, HbA1c and blood pressure were measured. Additionally, participants completed baseline questionnaires regarding their general health, physical activity, and dietary habits. All assessments were conducted at the obstetric clinic.

#### 2.3.2 Phase 1: regular diet (weeks 0–2)

After the completion of baseline measurements, participants were instructed to continue with their usual dietary regular habits. They also received routine antenatal care and dietary consultations from dietitians and healthcare workers. After 2 weeks of following their usual regular diet, participants returned to the clinic for another set of measurements including blood sugar and serum insulin levels—(at fasting, 1 h, 2 h). The purpose of this two-week (phase 1) was to observe any changes in blood sugar and serum insulin levels from baseline measurements in participants who followed their regular meal patterns.

#### 2.3.3 Phase 2: dietary intervention (weeks 2–4)

After the completion of phase 1 primary measurements, participants were instructed to follow a specific food-ordering regimen vegetables first, followed by proteins, and carbohydrates last for the next 2 weeks. This dietary intervention aimed to improve glycemic control by optimizing meal sequencing to slow carbohydrate absorption and reduce post-meal glucose spikes (12–14). Tailored diet plans were provided for both vegetarian and non-vegetarian participants. Throughout the intervention period, participants were regularly monitored by dieticians and healthcare workers via a mobile health application-Jotform installed on their phones, as well as during patient’s regular visits, to ensure adherence and address any issues. Using a mobile health application (Jotform), participants uploaded images of their meals after consuming each macronutrient (vegetables, proteins, and carbohydrates). This application allowed dietitians to monitor meal logs and provide feedback. Participants were trained in the use of the mobile app, which was available on both Android and iOS platforms. The application was designed with a simple user interface, enabling participants to easily log meals and communicate with dietitians. The application collected data on meal adherence, which was reviewed by dietitians, who provided guidance as needed. Participants were encouraged to maintain their usual physical activity levels throughout the study. The specific diet plans followed during the intervention period, including the detailed composition of vegetables, protein sources, and carbohydrate foods, are provided in Supplementary material. These plans were designed to optimize glycemic control and insulin sensitivity in participants, with a focus on food sequence: vegetables were consumed first, followed by protein, and carbohydrates were consumed last. A Comprehensive breakdown of energy, macronutrients, and food types, including these specific items, is provided in the Supplementary material, which aligns with the study’s objectives to improve dietary management in women with gestational diabetes.

#### 2.3.4 Follow-up measurement (week 4)

At the end of phase 2 (following 2 weeks of the dietary intervention—food order), participants consumed a meal (339 kcal, with 16.4 g protein, 56.1 g carbohydrates, and 3.4 g fats) in the prescribed food order, after a 12-h overnight fast, in the clinic. Blood glucose and serum insulin levels were measured again. Dietician recalled the participants’ nutritional intake during each phase of the study.

### 2.4 Study criteria

#### 2.4.1 Inclusion criteria

The inclusion criteria were as follows: the study participants should be (i) gestational diabetes women on lifestyle modification (diet + exercise); (ii) gestational weeks between 24–28 weeks; (iii) aged 18–40; (iv) signed an informed consent; (v) comfortable using mobile health application for their health management; (vi) participants who accept to adhere the food order and dietary plans.

#### 2.4.2 Exclusion criteria

Study participants were excluded if they were in any of these following criteria: (i) use of insulin therapy or any other blood glucose-lowering drugs (BGLDs), (ii) pre-existing type 1 or type 2 diabetes, (iii) gestational weeks above 28 or below 24 weeks, (iv) any severe medical conditions, (v) uncontrolled hypertension, severe food allergies or intolerances from specific dietary intervention, (vi) multiple gestations.

### 2.5 Patient consent

Patient consent was obtained and documented in accordance with protocols.

### 2.6 Mobile health application for dietary recall tracking

The dietary tracking and participant adherence to the food-ordering regimen were monitored through the Jotform mobile health application. Jotform is a standardized, user-friendly, form-building platform, widely used in clinical research for data collection (15). The application is based on JavaScript (React.js) for the front-end user interface, with PHP and MySQL for backend processing and data storage. It uses Amazon Web Services (AWS) for cloud hosting, ensuring scalability and reliability. This mobile application has been used in several recent studies for dietary tracking in clinical research settings, demonstrating its reliability and ease of use (16–18). This app allowed participants to log their meals by selecting meal types (e.g., breakfast, lunch, dinner), uploading images of their meals, and reporting the macronutrient. Participants were trained in an initial onboarding session, which included step-by-step guidance on how to log meals and take photos of their plates after consuming each macronutrient. Healthcare workers and dieticians were also trained on reviewing the meal logs and providing feedback to ensure compliance with the dietary intervention.

### 2.7 Measurement of outcomes

The primary outcomes of this study were blood glucose and serum insulin levels, measured three times: baseline (week 0), regular diet phase (weeks 0–2), and dietary intervention phase (weeks 2–4). Blood glucose levels were assessed at fasting, 1 h postprandial, and 2 h postprandial, and serum insulin levels were also measured at each time point to evaluate the effect of the dietary interventions on glycemic control and insulin resistance. Blood glucose was measured using the glucose oxidase enzymatic method, a reliable and widely used technique. The measurements were performed using the Beckman Coulter DxC 700 AU analyzer, ensuring accurate and reproducible results. Serum insulin levels were measured using the Enhanced Chemiluminescent Immunoassay (eCLIA), a highly sensitive and precise method, also conducted on the Beckman Coulter DxC 700 AU analyzer. Secondary outcomes, including cholesterol levels, HbA1c, body mass index (BMI), and blood pressure, were assessed only at baseline (week 0). Cholesterol levels (total cholesterol, LDL-c, HDL-c, and triglycerides) were measured using enzymatic assays. HbA1c, an important marker for long-term glycemic control, was measured using high-performance liquid chromatography (HPLC), which separates and quantifies HbA1c. BMI was calculated using the formula: weight (kg)/height (m^2^), and blood pressure was measured using a calibrated sphygmomanometer in a seated position after a five-minute rest, with two readings taken to ensure accuracy. These measurements provided valuable data for assessing the effects of dietary interventions on glycemic control, insulin sensitivity, and other key health markers related to gestational diabetes.

### 2.8 Statistical analysis

Statistical analysis was done using SPPS software, version 25. Data sets are represented as means ± standard deviation, *p*-values were calculated using the Wilcoxon matched-pairs signed rank test *p* < 0.01 was considered statistically significant.

## 3 Results

### 3.1 Nutritional differences between regular diet (phase 1) and food order (phase 2) diet

The analysis of nutritional intake before and after the dietary intervention (food order) revealed significant changes in macronutrient composition and food timing (Table 2). The food order diet resulted in a 10% reduction in total caloric intake, providing 1,700 kcal (±120 kcal), compared to 1,900 kcal (±150 kcal) in the regular diet. This reduction is largely due to the prioritization of vegetables and proteins, which naturally led to a decrease in calorie-dense foods such as carbohydrates and fats. There was a notable shift in carbohydrate consumption, with a 28% reduction in the food order diet, dropping from 250 g (±15 g) in the regular diet to 180 g (±12 g). This decrease can be attributed to the change in the timing of carbohydrate consumption, with carbohydrates eaten last, after vegetables and proteins, which may help in better blood sugar control. In contrast, the food order diet increases in other macronutrients, such as dietary fiber, which raised by 20%, from 25 g (±5 g) to 30 g (±6 g). This increase in fiber is likely due to a higher consumption of vegetables and fruits, which are rich in fiber and beneficial for digestion and blood sugar regulation. Protein intake also increased by 18%, rising from 55 g (±5 g) to 65 g (±7 g). The higher intake of protein-rich foods, such as dal, eggs, and chicken, helps improve satiety and supports glycemic control. Fat intake, however, decreased slightly by 9%, from 55 g (±8 g) to 50 g (±6 g), likely due to the increased intake of lean proteins and fiber-rich vegetables, which tend to be lower in fats. A significant change was observed in vegetable consumption, with an increase of 67%, from 150 g (±20 g) in the regular diet to 250 g (±30 g) in the food order diet. This increase is consistent with the dietary intervention, where vegetables were eaten first, leading to a higher intake of essential micronutrients such as vitamin C and folate, which support maternal and fetal health. Additionally, the timing of carbohydrate intake changed, with carbohydrates consumed last, after vegetables and proteins. This shift in food order is believed to help reduce postprandial glucose spikes, potentially improving blood sugar regulation and contributing to better overall glycemic control. These results suggest that the food order diet offers a more balanced nutritional profile, with higher fiber, protein, and vegetable intake, and a reduction in carbohydrate consumption. These changes could contribute to improved health outcomes, such as better blood sugar regulation, weight management, and metabolic control, highlighting the potential benefits of this dietary intervention for women with GDM.

**Table 2:** Comparison of nutritional intake before and after intervention in women with gestational diabetes mellitus (GDM) following regular diet and food order diet.

Nutrient	Regular diet	Food order diet	% change	Comments
Energy (kcal)	1,900 kcal (±150 kcal)	1,700 kcal (±120 kcal)	−10%	Energy intake reduced due to prioritizing vegetables and protein, lowering calorie-dense foods like carbs and fats
Fiber (g)	25 g (±5 g)	30 g (±6 g)	+20%	Increased fiber intake due to a higher consumption of vegetables and fruits, which improves digestion and blood sugar control
Proteins (g)	55 g (±5 g)	65 g (±7 g)	+18%	Higher intake of protein-rich foods (e.g., dal, eggs, chicken) helps in satiety and glycemic control
Fats (g)	55 g (±8 g)	50 g (±6 g)	−9%	Slight reduction in fats due to an increase in fiber-rich vegetables and lean proteins
Carbohydrates (CHO)	250 g (±15 g)	180 g (±12 g)	−28%	Reduction in carbohydrates due to consuming them last, after vegetables and proteins, which helps control glucose levels
Micronutrients	Varies (depends on food choices)	Higher (e.g., vitamin C, folate)	—	More vegetables and fruits lead to increased intake of essential micronutrients, supporting maternal and fetal health
Total vegetables (g)	150 g (±20 g)	250 g (±30 g)	+67%	Significant increase in vegetable consumption, as vegetables are eaten first in the food order diet
Carb timing	Mixed (throughout the meal)	Carbs eaten last after vegetables & protein	—	Consuming carbs last helps reduce postprandial glucose spikes, improving blood sugar regulation

### 3.2 Effect of food order on glucose and insulin levels

The analysis of glucose and insulin levels (Table 3) revealed that consuming vegetables before carbohydrates significantly reduced both postprandial blood glucose and serum insulin levels compared to regular diet. Also, there were no significant changes in blood glucose and serum insulin levels from baseline to the regular diet phase, as the measurements remained similar, indicating that continuing the regular diet did not induce any notable alterations in these parameters until the food order diet was implemented.

**Table 3:** Glucose and insulin levels, along with iAUC, were measured at various time points during the three visits.

GDM (25)	Time (mins)	Baseline measurements (0 weeks)	Measurements at 2nd weeks (regular diet)	Measurements at 4th week (food order diet)	*p* ^c^	Change (%)
Blood glucose (mg/dl)^a^	0	100.68 ± 4.6	100.76 ± 4.2	98.76 ± 5	0.18	−1.98
	60	148.72 ± 4.3	149.88 ± 4.6	141.08 ± 5.2	0.001	−5.87
	120	130.76 ± 3.4	130.08 ± 3.8	122.2 ± 4.6	0.001	−6.06
Serum insulin (μIU/mL)^a^	0	17.61 ± 0.8	17.68 ± 0.8	17.2 ± 0.8	0.021	−2.71
	60	78.46 ± 6.8	80.2 ± 2.7	73.68 ± 3.4	0.001	−8.13
	120	58.4 + 5 ± 5.3	60.56 ± 3.7	53.84 ± 7.1	0.001	−11.10
Glucose iAUC (mg/dL × min)^b^	0–60	1441.2 ± 209.89	1473.6 ± 210.05	1269.6 ± 209.05	0.04	−13.84
	0–120	3784.7 ± 573.83	3826.8 ± 579.27	3242.4 ± 503.63	0.03	−15.27
Insulin iAUC (μIU/mL × min)^b^	0–60	1825.5 ± 92.66	1875.6 ± 82.66	1,694 ± 96.48	<0.01	−9.66
	0–120	4876.1 ± 238.91	5037.6 ± 246.91	4,488 ± 304.34	<0.01	−10.91

Data are means ± standard deviation, *n* = 25.

a Blood samples were collected right before the meal (*t* = 0 min) and again at 60 and 120 min after the start of the meal.

b Intervals were measured in minutes from the start of the meal.

c *p*-values were calculated using the Wilcoxon matched-pairs signed rank test for 2nd (regular diet) and 4th week (food order diet) measurements. Change (%) were calculated for 2nd and 4th week measurements.

The use of the incremental area under the curve (iAUC) test highlighted the overall glucose and insulin response to a meal or glucose challenge over time. The results demonstrated a clear improvement in glycemic control and insulin sensitivity, suggesting a beneficial effect for managing GDM.

#### 3.2.1 Blood glucose levels

The mean blood glucose levels were significantly reduced by 5.87% (*p* = 0.001) at 60 min and by 6.06% (*p* = 0.001) at 120 min following the food order diet compared to the regular diet, demonstrating improved glucose regulation. The iAUC for glucose over 60 min (iAUC₀₋₆₀) was 13.84% lower (1269.6 ± 209.05 vs. 1473.6 ± 210.05 mg/dL × min, *p* = 0.04), and the iAUC₀₋₁₂₀ was 15.27% lower (3242.4 ± 503.63 vs. 3826.8 ± 579.27 mg/dL × min, *p* = 0.03).

#### 3.2.2 Serum insulin levels

Serum insulin levels also showed a significant decrease, dropping by 8.13% (*p* = 0.001) at 60 min and by 11.10% (*p* = 0.001) at 120 min with the food order diet compared to regular diet. The iAUC for insulin over 60 min (iAUC₀₋₆₀) was 9.66% lower (1,694 ± 96.48 vs. 1875.6 ± 82.66 μIU/mL × min, *p* < 0.01), and the iAUC₀₋₁₂₀ was 10.91% lower (4,488 ± 304.34 vs. 5037.6 ± 246.91 μIU/mL × min, *p* < 0.01).

These findings suggest that the sequence of food consumption, specifically eating vegetables before carbohydrates, significantly enhances metabolic control. The observed reduction in serum insulin levels indicates improved insulin sensitivity. Lower insulin levels mean that the body requires less insulin to maintain normal blood sugar levels. This reduction can help control diabetes by preventing insulin resistance, which is a hallmark of T2DM. Improved insulin sensitivity allows the body’s cells to respond more effectively to insulin, enhancing glucose uptake and regulation. Ultimately, this may help manage blood sugar levels more effectively and potentially reduce the need for exogenous insulin. This improved glucose regulation could contribute to better management of blood glucose levels and may reduce the long-term risk of developing T2DM.

## 4 Discussion

This study demonstrates that consuming vegetables first, followed by proteins, and carbohydrates last, significantly enhances glycemic control and insulin sensitivity in women with GDM, while continuing a regular diet showed no significant changes from baseline measurements. This highlights how even a small change in food sequence can have a notable impact. These findings are consistent with previous research on the effects of food order on postprandial glucose levels, contributing to our understanding of dietary strategies for blood sugar management. A pilot study by Shukla et al. (7) showed that consuming vegetables and proteins before carbohydrates resulted in significant reductions in postmeal glucose and insulin levels among 11 subjects with T2DM who consumed the same meal composition on 2 days, one-week apart. Specifically, mean postmeal glucose levels decreased by 28.6% (*p* = 0.001), 36.7% (*p* = 0.001), and 16.8% (*p* = 0.03) at 30, 60, and 120 min, respectively. The incremental area under the curve (iAUC_0–120_) was 73% lower when vegetables and protein were consumed first, compared to the reverse order. These results underscore the importance of food order as a practical dietary intervention, potentially enhancing glycemic control and insulin sensitivity. Although, this study focused on a small sample of T2DM subjects, our research expands on this by applying a similar dietary intervention to a larger cohort of 25 women with GDM. Variations in outcomes may arise from differences in sample size, the distinct physiological characteristics of GDM subjects compared with T2DM, the time interval between each test or differences in meal composition. Shukla et al. (11) also tested 16 T2DM subjects using the same meal composition on 3 days in random order. When carbohydrates were consumed last, both incremental areas under the curve for glucose (iAUC_0–180_) and incremental glucose peaks levels decreased significantly (by 53 and 54%), insulin excursions dropped, and GLP-1 levels increased, suggesting that eating carbohydrates last in a meal pattern may effectively improve postprandial glycemia compared to consuming carbohydrates first or having all meal components together. Furthermore, Imai et al. (19) found that consuming a meal with vegetables first significantly improved postprandial blood glucose and insulin levels in 18 healthy young women, regardless of eating speed. Both fast and slow eating regimens with vegetables prioritized yielded better outcomes than slow eating with carbohydrates first, underscoring the importance of food order. The research findings of our current work are well corroborated with the research findings of Shukla et al. (7, 11) and Imai et al. (19). Yong et al. (20) conducted a study on nutrient sequences involving 10 women with GDM who consumed the same caloric foods in different sequences over 5 days. They concluded that the most significant glycemic excursions occurred when carbohydrates were consumed first, suggesting that modifying meal sequences could help better manage blood sugar levels in GDM. Guo et al. (21) conducted their study of 124 women with GDM found that using a mobile health app improves patient compliance and blood glucose control, reduces weight gain, and consequently lowers the rates of complications for both pregnant women and their fetuses during delivery. Supporting evidence from previous research highlights that prioritizing vegetables and proteins before carbohydrates can significantly enhance dietary strategies. Furthermore, effective interventions, such as dietary counseling and mobile health apps, play a crucial role in managing GDM and reducing associated complications for both mothers and their infants.

## 5 Limitations and future directions

While this study offers valuable insights, several limitations should be acknowledged. The sample size of 25 participants may restrict the generalizability of the findings. Future research should involve larger and more diverse populations to confirm these results. Furthermore, the short duration of the intervention limits our understanding of long-term adherence to this dietary strategy and its sustained effects on glycemic control and insulin sensitivity. Dietitians also faced challenges in guiding participants through the mobile health application, particularly as many women experienced vomiting, which led to skipped meals. Although the dietary recall process using the application facilitated easier tracking and personalized feedback, issues related to data accuracy, technology use, and privacy remain crucial considerations. Longitudinal studies are essential to evaluate the impact of the food order approach over time and its potential influence on the long-term risk of developing T2DM.

## 6 Conclusion

In conclusion, the practice of food order, where vegetables are consumed first, followed by proteins and carbohydrates, significantly improves blood glucose and insulin levels in women with GDM. Our findings demonstrate that this simple dietary strategy enhances postprandial glycemic control, promoting better blood sugar regulation and potentially reducing the long-term risk of developing T2DM. The observed reduction in serum insulin levels at each time point suggests improved insulin sensitivity, likely due to the lower postprandial glucose response when vegetables are consumed first. This improvement in insulin sensitivity may help manage GDM more effectively and support better maternal health outcomes. Encouraging the increased intake of vegetables not only improves glycemic control but also promotes higher fiber consumption and reduces reliance on high-carbohydrate foods. Focusing on the sequence of food intake rather than solely on total energy consumption provides a practical and easily teachable method for healthcare professionals, making it a promising approach for managing GDM. Furthermore, this strategy offers an accessible intervention that could be incorporated into clinical practice to help reduce the risk of T2DM in the future. While this study highlights the benefits of food order in GDM, further research is essential to explore its long-term effects and broader applications, particularly in terms of maternal and fetal health. The simplicity of the intervention, coupled with its potential impact, makes it an exciting avenue for improving outcomes in women with GDM.

## Data Availability

The raw data supporting the conclusions of this article will be made available by the authors, without undue reservation.
