# Supplementary material for: Food order affects blood glucose and insulin levels in women with gestational diabetes
Source: Front Nutr. 2024 Dec 24;11:1512231. doi: 10.3389/fnut.2024.1512231 (PMC11703717; doi:10.3389/fnut.2024.1512231)
Supplement: Supplementary file 1 [file Table_1.docx]

| **DIET PLAN FOR GESTATIONAL DIABETES MELLITUS** | | | | | | | |
| --- | --- | --- | --- | --- | --- | --- | --- |
|  |  |  |  |  |  |  |  |
| **VEGETARIAN DIET PLAN** | | | | | | | |
| **MEAL TIMINGS** | **FOOD ITEMS** | **QUANTITY** | **ENERGY (kCal)** | **PROTEIN (g)** | **CARBOHYDRATES (g)** | **FAT (g)** | **FIBRE (g)** |
| **EARLY MORNING**  **06.00 AM – 07.00 AM** | Milk without Sugar/Jaggery/Brown sugar | 100ml | 67 | 3.2 | 4.4 | 4.1 | 0 |
|  |  |  |  |  |  |  |  |
| **BREAKFAST**  **08.00 AM – 09.00 AM** | Chutney – Ridge gourd / Bottle gourd or Thick dhal (Moong dal) /Sambar (Thoor dal) | 50g | 31-38 | 0.2-2.5 | 1.5-6.3 | 0.4-2.8 | 0.9-1.2 |
|  | Citrus fruit juice (Lemon/ Mosambi / Orange) without sugar  (Add 1 tsp drumstick leaves powder on top of juice just before consumption) | 100ml | 38-45 | 0.7 | 0.2-10.3 | 0 | 0 |
|  | Drumstick leaves powder | 1 Tsp | 8 | 0.9 | 0.7 | 0.2 | 1.6 |
|  | Pongal - Little Millet/Broken Wheat or | 150g | 113-199 | 2.9-7.3 | 16.5-27.1 | 1.3-10.9 | 1.6-5.2 |
|  | Upma - Broken Wheat or |  |  |  |  |  |  |
|  | Idli - Finger millet/ Pearl millet or | 2nos (100g) |  |  |  |  |  |
|  | Adai - Foxtail millet/ Pearl millet/ Finger millet/ Sorghum or |  |  |  |  |  |  |
|  | Dosa - Finger millet/ Pearl millet |  |  |  |  |  |  |
|  |  |  |  |  |  |  |  |
| **MID-MORNING**  **11.00 AM – 12.00 PM** | Fruits – Apple / Oranges / Pomegranate / Guava | 100g | 48-83 | 0.2-2.6 | 10.9-18.7 | 0.2-1.2 | 1.1-5.4 |
|  | Cucumber | 100g | 15 | 0.7 | 3.6 | 0.1 | 0.5 |
|  | Nuts - Almonds/Walnuts | 10nos(12g/42g) | 74-282 | 2.5-6.3 | 2.1-4.3 | 6.0-27 | 1.1-2.3 |
|  |  |  |  |  |  |  |  |
| **LUNCH**  **01.00 PM – 02.00 PM** | Vegetable poriyal/kootu - ladies' finger, green beans, cabbage, green peas, brinjal, cauliflower | 75g | 68 | 1.9 | 8 | 3.3 | 2.8 |
|  | Greens kootu/poriyal (Squeeze 1 tsp lemon juice on top of greens poriyal just before consumption) | 75g | 60 | 2.5 | 5.5 | 3.1 | 2.1 |
|  | Lemon juice | 1 tsp | 1 | 0 | 0.4 | 0 | 0 |
|  | Sambar (Thoor dal) | 100ml | 76 | 3.7 | 108 | 2 | 2.5 |
|  | White rice/brown rice/little millet/ Kodo millet/ Pearl millet (cooked using straining method) | 100g | 76-103 | 1.9-3.3 | 14.3-21.5 | 0.3-1.4 | 0.4-3.2 |
|  | Curd | 100g | 60 | 3.1 | 3 | 4 | 0 |
|  |  |  |  |  |  |  |  |
| **EVENING SNACKS**  **04.00 PM – 05.00 PM** | Steamed sundals or Steamed sprouts or Steamed sorghum | 100g | 92-155 | 2.8-9.9 | 17.2-26.6 | 0.5-1.4 | 2.4-9.6 |
|  | Milk without Sugar/Jaggery/Brown sugar | 100ml | 67 | 3.2 | 4.4 | 4.1 | 0 |
|  |  |  |  |  |  |  |  |
| **DINNER**  **06.30 PM – 07.00 PM** | Chutney – Ridge gourd / Bottle gourd /Mint chutney/tomato chutney or Thick dhal (Moong dal) /Sambar (Thoor dal) | 50g | 15-38 | 0.2-2.5 | 1.5-6.3 | 0.1-2.8 | 0.9-1.7 |
|  | Paneer bhurji (without oil) | 100g | 122 | 7.6 | 5.3 | 7.9 | 1.6 |
|  | Kitchadi - Broken wheat / Kodo millet / Little millet or | 150g | 111-174 | 2.7-7.1 | 16.7-34.6 | 1.0-4.5 | 2.9-6.6 |
|  | Idiyappam - Finger millet or |  |  |  |  |  |  |
|  | Multigrain fenugreek leaves chappati | 2nos (80g) |  |  |  |  |  |
| **BEDTIME**  **08.30 PM – 09.00 PM** | Milk without Sugar/Jaggery/Brown sugar | 100ml | 67 | 3.2 | 4.4 | 4.1 | 0 |
|  |  |  |  |  |  |  |  |
| **TOTAL** |  |  | **1209-1727** | **44.1-72.2** | **228.6-303** | **42.7-84.9** | **22.4-46.3** |
| **REQ** |  |  | **1725** | **65** | **259** | **48** | **28** |

| **NON-VEGETARIAN DIET PLAN** | | | | | | | |
| --- | --- | --- | --- | --- | --- | --- | --- |
| **MEAL TIMINGS** | **FOOD ITEMS** | **QUANTITY** | **ENERGY (kCal)** | **PROTEIN (g)** | **CARBOHYDRATES (g)** | **FAT (g)** | **FIBRE (g)** |
| **EARLY MORNING**  **06.00 AM – 07.00 AM** | Milk without Sugar/Jaggery/Brown sugar | 100ml | 67 | 3.2 | 4.4 | 4.1 | 0 |
|  |  |  |  |  |  |  |  |
| **BREAKFAST**  **08.00 AM – 09.00 AM** | Chutney – Ridge gourd / Bottle gourd or Thick dhal (Moong dal) /Sambar (Thoor dal) | 50g | 31-38 | 0.2-2.5 | 1.5-6.3 | 0.4-2.8 | 0.9-1.2 |
|  | Egg white | 1no | 17 | 3.6 | 0.2 | 0.2 | 0 |
|  | Buttermilk | 100ml | 18 | 0.9 | 1 | 1.2 | 0 |
|  | Pongal - Little Millet/Broken Wheat or | 150g | 113-199 | 2.9-7.3 | 16.5-27.1 | 1.3-10.9 | 1.6-5.2 |
|  | Upma - Broken Wheat or |  |  |  |  |  |  |
|  | Idli - Finger millet/ Pearl millet or | 2nos (100g) |  |  |  |  |  |
|  | Adai - Foxtail millet/ Pearl millet/ Finger millet/ Sorghum or |  |  |  |  |  |  |
|  | Dosa - Finger millet/ Pearl millet |  |  |  |  |  |  |
| **MID-MORNING**  **11.00 AM – 12.00 PM** | Fruits – Apple / Oranges / Pomegranate / Guava | 100g | 48-83 | 0.2-2.6 | 10.9-18.7 | 0.2-1.2 | 1.1-5.4 |
|  | Cucumber | 100g | 15 | 0.7 | 3.6 | 0.1 | 0.5 |
|  | Nuts - Almonds/Walnuts | 10nos(12g/42g) | 74-282 | 2.5-6.3 | 2.1-4.3 | 6.0-27 | 1.1-2.3 |
|  |  |  |  |  |  |  |  |
| **LUNCH**  **01.00 PM – 02.00 PM** | Vegetable poriyal/kootu - ladies' finger, green beans, cabbage, green peas, brinjal, cauliflower | 75g | 68 | 1.9 | 8 | 3.3 | 2.8 |
|  | Greens kootu/poriyal - Squeeze 1 tsp lemon juice on top of greens poriyal just before consumption | 75g | 60 | 2.5 | 5.5 | 3.1 | 2.1 |
|  | Lemon juice | 1 tsp | 1 | 0 | 0.4 | 0 | 0 |
|  | Sambar (Thoor dal) or | 100ml | 76-105 | 3.7-11.4 | 3.5-108 | 2-5.3 | 1.2-2.5 |
|  | Fish curry (TO AVOID - shark, swordfish, king mackerel, tile fish because of high mercury content) or |  |  |  |  |  |  |
|  | Chicken breast curry |  |  |  |  |  |  |
|  | Steamed fish or egg white | 2nos (160g) | 34-148 | 7.2-25.4 | 0.4-6.7 | 0.4-2.1 | 0 |
|  | White rice/brown rice/little millet/ Kodo millet/ Pearl millet (cooked using straining method) | 100g | 76-103 | 1.9-3.3 | 14.3-21.5 | 0.3-1.4 | 0.4-3.2 |
|  |  |  |  |  |  |  |  |
| **EVENING SNACKS**  **04.00 PM – 05.00 PM** | Steamed sundals or Steamed sprouts or Steamed sorghum | 100g | 92-155 | 2.8-9.9 | 17.2-26.6 | 0.5-1.4 | 2.4-9.6 |
|  | Milk without Sugar/Jaggery/Brown sugar | 100ml | 67 | 3.2 | 4.4 | 4.1 | 0 |
|  |  |  |  |  |  |  |  |
| **DINNER**  **06.30 PM – 07.00 PM** | Chutney – Ridge gourd / Bottle gourd /Mint chutney/tomato chutney or Thick dhal (Moong dal) /Sambar (Thoor dal) | 50g | 15-33 | 0.2-1.4 | 1.5-5.4 | 0.1-2.8 | 1.0-1.7 |
|  | Egg white bhurji | 100g | 83 | 5.7 | 3.7 | 5 | 1.1 |
|  | Kitchadi - Broken wheat / Kodo millet / Little millet or | 150g | 111-174 | 2.7-7.1 | 16.7-34.6 | 1.0-4.5 | 2.9-6.6 |
|  | Idiyappam - Finger millet or |  |  |  |  |  |  |
|  | Multigrain fenugreek leaves chappati | 2nos (80g) |  |  |  |  |  |
| **BEDTIME**  **08.30 PM – 09.00 PM** | Milk without Sugar/Jaggery/Brown sugar | 100ml | 67 | 3.2 | 4.4 | 4.1 | 0 |
|  |  |  |  |  |  |  |  |
| **TOTAL** |  |  | **1133-1782** | **49.2-102.1** | **120.2-294.4** | **37.4-84.6** | **19-44.2** |
| **REQ** |  |  | **1725** | **65** | **259** | **48** | **28** |

**Reference**

1. Atkinson FS. 2008. International Tables of Glycemic Index and Glycemic Load Values: 2008. *Diabetes Care*. 31(12):2281-2283. doi: 10.2337/dc08-1239.
2. Sabarathinam S. 2023. A glycemic diet improves the understanding of glycemic control in diabetes patients during their follow-up. *Future Science OA*. Available online.
3. Derbo ZD. 2023. The effect of fresh moringa leaf consumption during pregnancy on maternal hemoglobin level in Southern Ethiopia: Multilevel analysis of a comparative cross-sectional study. *Int J Womens Health*. 15:243-255. doi: 10.2147/IJWH.S436123.
4. Sabarathinam S. 2023. A glycemic diet improves the understanding of glycemic control in diabetes patients during their follow-up. *Future Science OA*. Available online.
5. Sharmin R. 2013. Hypoglycemic and hypolipidemic effects of cucumber, white pumpkin, and ridge gourd in alloxan-induced diabetic rats. *J Sci Res*. 5(1):55-60. doi: 10.3329/jsr.v5i1.13290.
6. Brown R. 2021. Snacking on almonds lowers glycaemia and energy intake compared to a popular high-carbohydrate snack food: An acute randomized crossover study. *Int J Environ Res Public Health*. 18(8):4154. doi: 10.3390/ijerph18084154.
7. Sahariah SA. 2016. A daily snack containing leafy green vegetables, fruit, and milk before and during pregnancy prevents gestational diabetes in a randomized, controlled trial in Mumbai, India. *J Nutr*. 146(10):1890-1898. doi: 10.3945/jn.116.237933.
8. Shakappa D. 2022. Glycemic carbohydrates, glycemic index, and glycemic load of commonly consumed South Indian breakfast foods. *J Food Sci Technol*. 59(6):2561-2570. doi: 10.1007/s11483-022-05602-0.
9. Shobana S. 2022. Carbohydrate profiling & glycaemic indices of selected traditional Indian foods. *Indian J Med Res*. 155(3):336-343. doi: 10.4103/ijmr.IJMR_1716_20.
10. Sabarathinam S. 2023. A glycemic diet improves the understanding of glycemic control in diabetes patients during their follow-up. *Future Science OA*. Available online.
11. Kumar V. 2011. Comparative study of fenugreek seeds on glycemic index in high and medium dietary fiber containing diets in NIDDM patients. *ResearchGate*. Available online.
12. Pérez-Roncero GR. 2020. The effect of consuming milk and related products during human pregnancy over birth weight and perinatal outcomes: A systematic review and meta-analysis. *Eur J Obstet Gynecol Reprod Biol*. 253:14-24. doi: 10.1016/j.ejogrb.2020.01.004.
13. Bramante CT. 2018. Fish consumption during pregnancy: An opportunity, not a risk. *JAMA Pediatr*. 172(6):533-534. doi: 10.1001/jamapediatrics.2018.0611.
14. Beasant L. 2023. Fish consumption during pregnancy in relation to national guidance in England in a mixed-methods study: The PEAR study. *Nutrients*. 15(9):2123. doi: 10.3390/nu15092123.
15. Shakappa D. 2022. Glycemic carbohydrates, glycemic index, and glycemic load of commonly consumed South Indian breakfast foods. *J Food Sci Technol*. 59(6):2561-2570. doi: 10.1007/s11483-022-05602-0.
16. Shobana S. 2022. Carbohydrate profiling & glycaemic indices of selected traditional Indian foods. *Indian J Med Res*. 155(3):336-343. doi: 10.4103/ijmr.IJMR_1716_20.
17. Sabarathinam S. 2023. A glycemic diet improves the understanding of glycemic control in diabetes patients during their follow-up. *Future Science OA*. Available online.
18. Kumar V. 2011. Comparative study of fenugreek seeds on glycemic index in high and medium dietary fiber containing diets in NIDDM patients. *ResearchGate*. Available online.
19. Pérez-Roncero GR. 2020. The effect of consuming milk and related products during human pregnancy over birth weight and perinatal outcomes: A systematic review and meta-analysis. *Eur J Obstet Gynecol Reprod Biol*. 253:14-24. doi: 10.1016/j.ejogrb.2020.01.004.
